# Advances in Biosensors for the Rapid Detection of Marine Biotoxins: Current Status and Future Perspectives

**DOI:** 10.3390/bios14040203

**Published:** 2024-04-19

**Authors:** Xiangwei Zhu, Yufa Zhao, Long Wu, Xin Gao, Huang Huang, Yu Han, Ting Zhu

**Affiliations:** 1 National “111” Center for Cellular Regulation and Molecular Pharmaceutics, Key Laboratory of Fermentation Engineering (Ministry of Education), Hubei Key Laboratory of Industrial Microbiology, Hubei University of Technology, Wuhan 430068, China; xiangwei@ksu.edu (X.Z.); c13886776274@163.com (Y.Z.); huanghuang@hbut.edu.cn (H.H.); zhuting066@hotmail.com (T.Z.); 2 School of Food Science and Engineering, Key Laboratory of Tropical Fruits and Vegetables Quality and Safety for State Market Regulation, Hainan University, Haikou 570228, China; dukadifalali@163.com; 3 Hubei Key Laboratory of Quality Control of Characteristic Fruits and Vegetables, College of Life Sciences and Technology, Hubei Engineering University, Xiaogan 432000, China

**Keywords:** food safety, surface-enhanced Raman spectroscopy, marine biotoxins, biosensing technology, limitations and prospects

## Abstract

Marine biotoxins (MBs), harmful metabolites of marine organisms, pose a significant threat to marine ecosystems and human health due to their diverse composition and widespread occurrence. Consequently, rapid and efficient detection technology is crucial for maintaining marine ecosystem and human health. In recent years, rapid detection technology has garnered considerable attention for its pivotal role in identifying MBs, with advancements in sensitivity, specificity, and accuracy. These technologies offer attributes such as speed, high throughput, and automation, thereby meeting detection requirements across various scenarios. This review provides an overview of the classification and risks associated with MBs. It briefly outlines the current research status of marine biotoxin biosensors and introduces the fundamental principles, advantages, and limitations of optical, electrochemical, and piezoelectric biosensors. Additionally, the review explores the current applications in the detection of MBs and presents forward-looking perspectives on their development, which aims to be a comprehensive resource for the design and implementation of tailored biosensors for effective MB detection.

## 1. Introduction

The ocean, a vast and complex natural ecosystem, is pivotal in addressing global challenges such as population growth, resource scarcity, and energy demands, owing to its abundant mineral and biological resources. It plays a crucial role in ensuring food safety, nutrition, health, and fostering social and economic development [1]. In recent years, the contamination of marine environments with biotoxins has become a significant concern [2]. Originating mainly from algae, phytoplankton, or microorganisms, these toxins can accumulate in marine species like shellfish and fish, persisting over time. Despite their resilience to standard processing techniques, including heating and microwaving, these toxins pose severe health risks when ingested by humans, affecting vital organs and systems, and potentially leading to poisoning incidents [3,4]. Consequently, it is paramount to develop detection techniques for the accurate and quantitative detection of these microbial toxins.

Annually, over 20,000 cases of food poisoning worldwide are attributed to marine biotoxins (MBs), with consequential risks to marine mammal populations [5]. Traditional detection methods for MBs, such as the mouse bioassay [6], high-performance liquid chromatography (HPLC) [7], liquid chromatography–mass spectrometry (LC-MS) [8,9], and enzyme-linked immunosorbent assay (ELISA) [10], vary in detection effectiveness and efficiency. For instance, the mouse bioassay provides a comprehensive means to evaluate the toxicological impacts of toxins, yet its sensitivity is low and the detection timeline is lengthy, rendering it suboptimal for swift, extensive field applications. While HPLC and LC-MS deliver precise detection, they are hampered by their high cost, significant equipment size, extended analysis duration, and the requirement for specialized expertise, limiting their utility in field analysis. Conversely, ELISA is noted for its rapidity and specificity, yet its conventional applications are not well-suited for detecting small molecules like MBs, given its relatively low sensitivity and reliance on a singular detection mechanism, typically antibody–antigen interactions. These limitations underscore the necessity for developing more effective detection methods for MBs.

Biosensors, engineered as methods and devices that specifically detect target substances and convert their concentrations into measurable optical, electrical, or mass signals via biological components, represent a promising technological solution [11]. Biosensors stand out as a practical choice for detecting MBs due to their straightforward design, rapid performance, and suitability for field use. Their flexible construction and high sensitivity, which can detect concentrations as low as picomoles per liter, further enhance their viability for this purpose. Currently, three types of biosensors are employed for this purpose: (1) optical biosensors, which include fluorescent [12,13], surface-enhanced Raman scattering (SERS) [14,15], colorimetric [16,17,18], and surface plasmon resonance (SPR) biosensors [19,20]; (2) electrochemical biosensors, such as voltammetric and impedance biosensors [21,22]; and (3) piezoelectric biosensors, exemplified by quartz crystal biosensors [23]. These biosensors, each based on distinct operating principles, are continuously refined and optimized to effectively align with the dynamic requirements of MBs detection.

**Figure 1:**
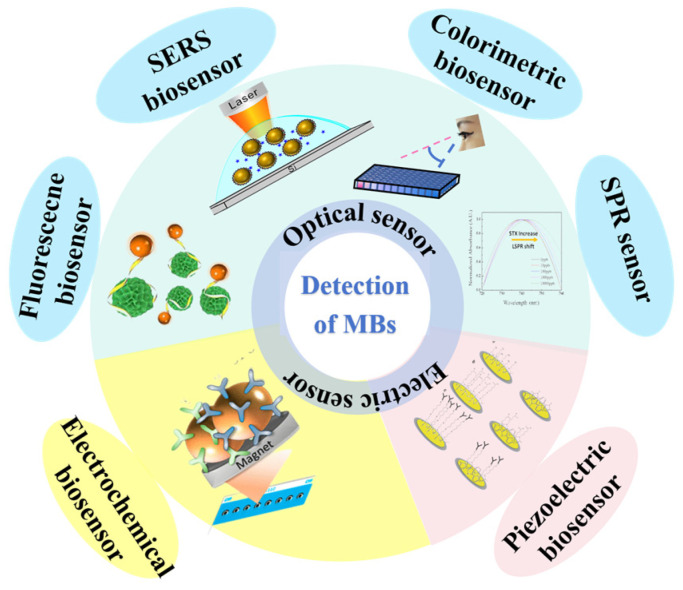
Classification of different biosensors for the detection of MBs.

This review systematically classifies biosensors into three distinct categories based on their signaling mechanisms: optical, electrochemical, and piezoelectric, as depicted in Figure 1. As a result, it provides thorough insights into the various application contexts, enumerating the benefits and limitations inherent to each type of biosensor. The objective is to offer a concise yet comprehensive evaluation of biosensing technologies’ efficacy and practicality, particularly highlighting recent advancements in detecting MBs. By doing so, the review aims to deepen the understanding of the intricate interplay between the ongoing development of detection technologies and their application in the field, thereby elucidating the dynamic nexus between technological innovation and its deployment in research settings.

## 2. Types and Sources of MBs

MBs are a diverse group of natural compounds produced by various marine organisms, including algae, bacteria, and invertebrates. These biotoxins can cause harm to human health, marine life, and ecosystems. Typically, they are classified based on their chemical structure, origin, and mode of action. The primary classification method is chemical structure, which divides biotoxins into three main types: peptide toxins, polyether toxins, and alkaloid toxins (Table 1) [24,25,26].

Peptide toxins are a type of small organic compound composed of multiple amino acid molecules, categorized by their chemical structure. Numerous peptide toxins have been identified, with extensively studied examples including conotoxin, sea anemone peptide toxin, and sea snake toxin. Conotoxin, derived from the venom of the tropical marine mollusk *Conus vulgaris*, is a compact neurotoxin rich in disulfide bonds. Conotoxins exhibit remarkable diversity and are classified into various types, such as α-conotoxin, μ-conotoxin, ω-conotoxin, σ-conotoxin, k-conotoxin, and λ-conotoxin, based on their specific neuromuscular targets [27]. Anemone polypeptide toxin, sourced from the venom of sea anemones’ cnidocysts, is another neurotoxin. These toxins can be categorized based on their respective targets, including voltage-gated Na^+^, voltage-gated K^+^ channels, and other ion channels. Sea snake toxins encompass enzymes, polypeptides, and small peptides secreted by sea snakes, with polypeptide toxins being the predominant components, recognized as postsynaptic neurotoxins or α-neurotoxins [28].

Polyether toxins are organic compounds characterized by multiple oxygenated ether rings and a high proportion of heteroatoms to carbon atoms. They are primarily classified into three groups: trapezoidal polyether toxins, linear polyether toxins, and macrolide polyether toxins [31]. Notable examples include ciguatoxin (CTX), palytoxin (PTX), and brevetoxin (BTX). CTX, a trapezoidal polyether toxin, is highly toxic and originates from the dinoflagellate *Gambierdiscus toxicus*. It accumulates in fish through the food chain and poses a risk to human health upon consumption [32]. PTX, a linear polyether toxin found in palythoa, is one of the most toxic and complex compounds among non-peptide MBs [31]. BTX, a macrolide polyether toxin, is primarily produced by certain species of nudibranchs and poses significant health threats to both marine fish and humans [8].

**Figure 2:**
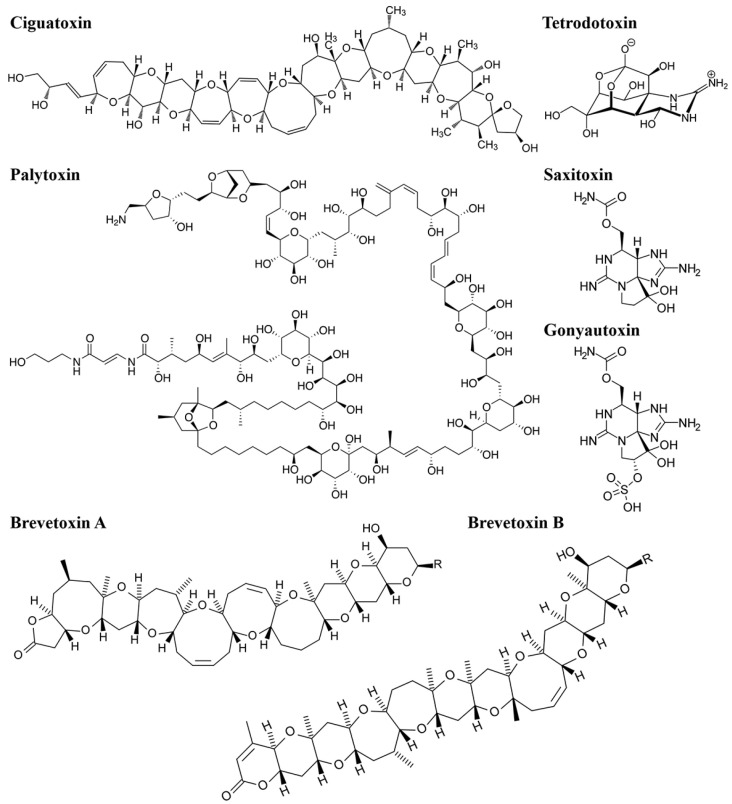
Structural formulae of six common MBs (CTX, PTX, BTX, TTX, STX, CTX).

Alkaloid toxins are nitrogen-containing compounds with complex carbon structures, often found as secondary metabolites in marine organisms. Common alkaloid toxins include tetrodotoxin (TTX), saxitoxin (STX), and gonyautoxin (GTX) (Figure 2). TTX, an amino pyrroloquinazoline-type neurotoxin, exists in puffer fish and other organisms, primarily paralyzing nerves and muscles. STX, initially discovered in clams and mussels, is recognized as one of the most potent MBs and functions as a guanidinoamine neurotoxin [29]. GTX is a guanidine neurotoxin produced by gondola and can be divided into GTX1, GTX2, GTX3, and GTX4 [33].

**Figure 3:**
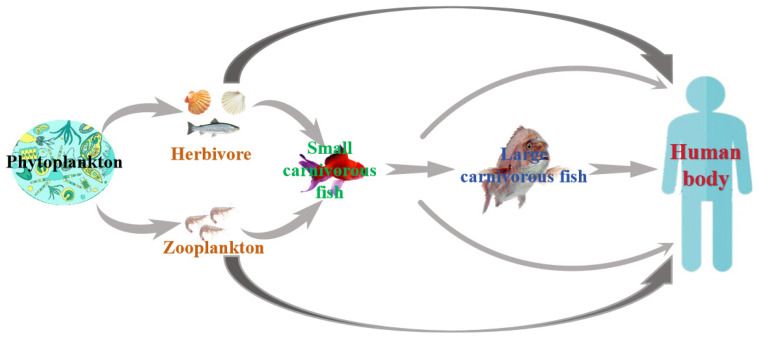
Scheme of enrichment and transmission of MBs through the food chain.

MBs primarily originate from marine microorganisms and algae, affecting a broad spectrum of species. These toxins can be categorized into two types: non-acquired and acquired. Non-acquired toxins are synthesized directly by the organisms themselves, mainly by marine bacteria, fungi, and algae. In contrast, acquired toxins are not produced by the organisms themselves but are accumulated through the food chain and environmental exposure. These toxins are predominantly found in fish, shellfish, and shrimp. MBs are known for their notable stability, persisting within marine organisms and their environments. As depicted in Figure 3, MBs in the marine environment are primarily produced by microorganisms and algae. These toxins accumulate in marine organisms either directly from the environment or through processes such as predation, parasitism, and symbiosis. Due to their relative stability, the accumulation of these toxins increases with each level of the marine food chain.

## 3. Hazards and Impacts of MBs

The hazards posed by MBs primarily center on their detrimental impacts on human and animal health, as well as their adverse effects on socio-economic sectors. When MB levels exceed safety thresholds, they can cause various health issues, including acute poisoning, chronic poisoning, allergic reactions, and even carcinogenic effects. These toxins typically exhibit high biological activity and target specific cells within organisms, primarily affecting the digestive, nervous, and cardiovascular systems. For instance, the ingestion of over 40.00 μg of okadaic acid (OA) may result in symptoms such as diarrhea, nausea, vomiting, abdominal pain, and chills in adults [34]. Although generally not life-threatening, OA is significantly associated with human esophageal cancer, gastric cancer, colon cancer, pancreatic cancer, and liver cancer. STX, although non-toxic to shellfish themselves, can cause the paralysis of limbs, headaches, fever, and potentially lead to respiratory failure, with a dose of 300.00 μg being fatal for adults [35]. On the other hand, CTX can inflict damage on the digestive, nervous, and cardiovascular systems, categorized into four levels of toxicity: acute, strong, mild, and slightly toxic [36]. 

The intake of MBs is correlates directly with the severity of human poisoning symptoms and may even cause irreversible injury or death. Given these severe consequences, there is heightened vigilance regarding marine biotoxin occurrences, which significantly impacts regional economic and industrial development. When MB levels exceed safety limits in algae, shellfish, shrimp, and fish, their sale is legally prohibited. Moreover, due to their high stability, removing these toxins through food processing techniques is challenging and economically unfeasible. Consequently, exceeding safe limits of MBs results in significant economic losses to aquaculture industries and can also have detrimental effects on tourism and the marine environment.

## 4. Development and Application of Biosensors

Ensuring the protection of human health and the marine ecosystem hinges significantly on the monitoring and detection of MBs. Currently, prevalent techniques for detecting these toxins encompass biological, physicochemical, and biosensor methods. Among these, biosensor detection stands out for its notable sensitivity and specificity. Biosensors, categorized based on their signal output methods, include optical, electrochemical, and piezoelectric variants. Optical biosensors are further subdivided into fluorescent, surface-enhanced Raman scattering (SERS), colorimetric, and surface plasmon resonance sensors. These distinctions allow for the precise and efficient detection of MBs, making biosensor methods particularly invaluable in safeguarding both human health and the marine environment.

### 4.1. Fluorescent Biosensors

Fluorescent biosensors employ a variety of signal sources, including fluorescent dyes, quantum dots (QDs), and nanomaterials, to detect MBs specifically through advanced techniques such as antigen-antibody recognition and aptamer binding. These biosensors capitalize on various biomolecular interactions to ensure precision. Noteworthy for their high sensitivity and rapid response times, they also boast high throughput capabilities and minimal background noise. Additionally, their design facilitates easy automation, making them ideal for streamlined, efficient monitoring and analysis in environmental and healthcare applications.

**Figure 4:**
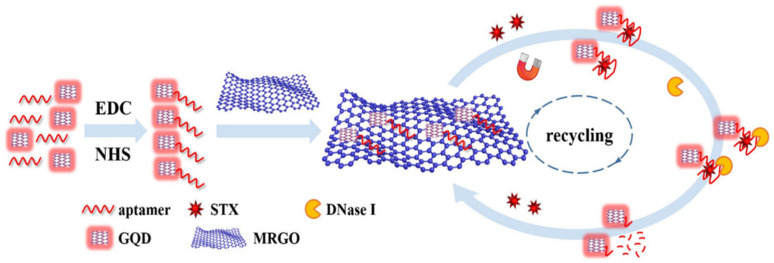
An aptamer-based fluorescent biosensor with nuclease-assisted target recycling signal amplification strategy and GQD fluorescence signal for detection of STX. Reproduced from [38] with permission from Springer Vienna.

Among fluorescent labeling materials, fluorescent dyes are commonly employed due to their diverse color range, intense fluorescence, and compatibility with biological molecules. For instance, Liu et al. developed a method using Cy3-labeled aptamers and AuNPs@MIL-101, achieving a tetrodotoxin detection range of 0.01~300.00 ng/mL with a limit of detection (LOD) as low as 6.00 pg/mL. QDs possess biocompatibility, high-fluorescence yields, and photostability, along with tunable emission spectra [37]. STX was detected using graphene quantum dots (GQDs) and nuclease-assisted target cycling signal amplification, as shown in Figure 4 [38]. The sensing mechanism involves the attachment of STX aptamer to the carboxyl group on the GQD surface via the amino group. Subsequently, the complex is adsorbed onto magnetized reduced graphene oxide (MRGO) through π-π stacking. During this process, the fluorescence is quenched by the MRGO. However, in the presence of STX, the STX aptamer–GQD complex dissociates from the MRGO surface, leading to the appearance of fluorescence signals. The detection range spans from 0.10 to 100.00 ng/mL, with an LOD as low as 0.035 ng/mL.

With the increasing need for multi-target detection, there is rising interest in employing labels characterized by high fluorescence efficiency and narrow emission spectra to reduce interference and boost detection precision. Recent advancements have been made in the introduction of lead-based chalcogenide nanomaterials with narrow photoluminescence spectra, indicating potential improvements in fluorescence emission efficiency and the broadening of applications for fluorescent biosensors [39]. In addition to biosensing, aptamer-based approaches prove valuable in toxicology studies and the analysis of marine products.

### 4.2. Surface-Enhanced Raman Scattering Biosensors

**Figure 5:**
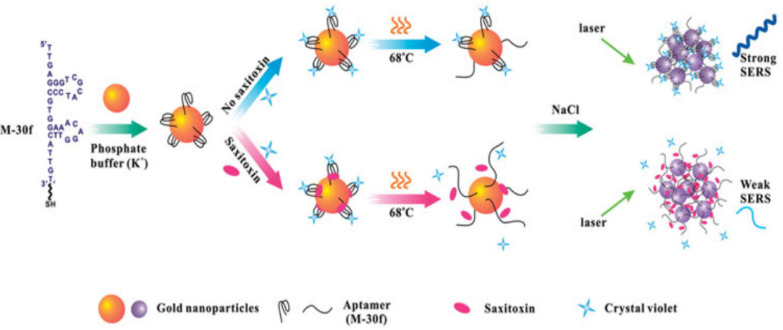
SERS aptamer sensing method for detection of STX based on Au NPs. Reproduced from [40] with permission from Taylor and Francis Ltd. (Oxford, UK).

A surface-enhanced Raman scattering (SERS) biosensor is a technology that utilizes the vibration of molecules on a brown metal surface or nanostructures to generate a unique fingerprint spectral signal intensity and thus determine the concentration of the molecules, which has the advantages of high sensitivity, low cost, and high speed. The Raman substrate and Raman signaling molecules are the main factors affecting the SERS signal. Among them, a high-performance Raman substrate should have a large area of high-density hotspots, excellent uniformity, good reproducibility, and a high enhancement factor. For example, Cheng et al. reported that gold nanoparticles (Au NPs) modified with STX aptamer (M-30f) were used as probes in SERS, as shown in Figure 5, with crystalline violet as the Raman signaling molecule, which detaches from the surface of Au NPs when STX is present, leading to weaker Raman signals, and its LOD for STX was 11.70 nM [40].

Similar to this, the utilization of a composite resonance bilayer consisting of uniformly dispersed Au NPs integrated with three-dimensional (3D) fleshy silver particles enabled multiple amplifications of SERS signals, achieving an LOD of 10 ng/mL for soft spongy acids [41]. The non-reproducibility of surface-enhanced Raman spectroscopy is the main limitation, and the adsorption of the target molecules with the surface-enhanced substrate cannot be controlled; also, the contact of biomolecules with metals may also destroy their surface Raman enhancement. Therefore, suitable functionalized SERS substrates are needed to regulate the adsorption of target molecules to the surface-enhanced substrates using electric or magnetic fields.

In addition, the performance of SERS largely depends on the quality of Raman substrates and signaling molecules. Optimal substrates feature densely packed hotspots and uniform, reproducible surfaces with high enhancement factors. Challenges include the non-reproducibility of Raman signals and potential damage to biomolecules upon metal contact, necessitating functionalized substrates for improved target molecule adsorption control.

### 4.3. Colorimetric Biosensors

**Figure 6:**
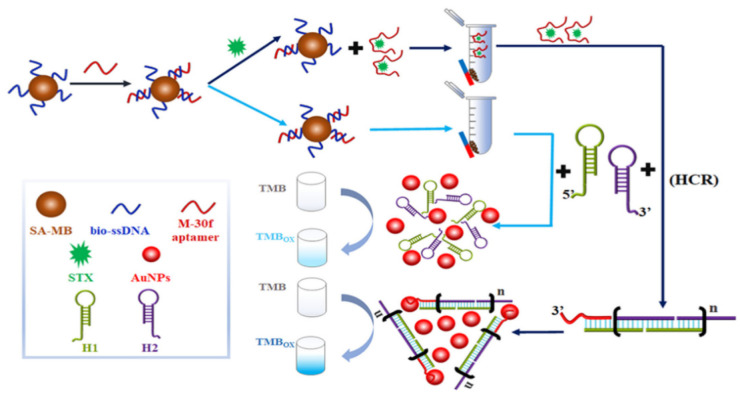
Competitive colorimetric biosensor induced by station change of Au NPs based on HCR mediation. Reproduced from [43] with permission from Elsevier.

Colorimetric biosensors are mainly used for qualitative and quantitative analyses based on nanoparticle morphology as well as changes in state, enzymes, and chemical reactions caused by color changes observed by the naked eye. Nanoparticles used for colorimetric biosensing mainly include Au NPs and silver nanomaterials (Ag NPs) in various shapes, and Au NPs are commonly used nanomaterials for colorimetric biosensors. For example, Qiang et al. designed a colorimetric biosensor based on aptamer modulation of the state of Au NPs, which specifically binds to STX, causing the aggregation of Au NPs and a change in the solution color from red to purple or blue, with an LOD of 3.00 fg/mL [42]. However, the Au NPs are subjected to interferences of pH, temperature, organic solvents, and the sample mechanism, and aggregation occurs, resulting in false-positive results. To reduce the likelihood of false positives in detection, researchers investigate the use of color changes induced by enzymes or nanoenzymes catalyzing substrates as detection indicators. For instance, a colorimetric aptamer biosensor for the sensitive detection of STX based on the hybridization chain reaction (HCR) is depicted in Figure 6. When STX is present, the aptamer separates from the magnetic beads, triggering the hybridization chain reaction through magnetically separated aptamers to form HCP-dsDNAs-Au. This significantly enhances the catalytic ability to induce color changes in TMB, thereby amplifying the signal specific to STX [43]. The LOD of this colorimetric aptamer sensor was 42.46 pM, with high sensitivity. With the development of smartphone image acquisition and processing technology, colorimetric biosensors have attracted widespread attention for the rapid detection of MBs.

Colorimetric biosensors utilize nanoparticle morphology and state changes for qualitative and quantitative analysis, primarily employing Au NPs or Ag NPs. These biosensors detect color shifts visible to the naked eye due to enzymatic or chemical reactions. For instance, changes in Au NP aggregation cause color transitions from red to purple, indicating specific analyte binding, though susceptible to environmental interferences. Advances include enzymatic reactions to enhance detection specificity and sensitivity, coupled with smartphone technology for rapid analysis, significantly improving the reliability and usability of these sensors in real-time applications.

### 4.4. Surface Plasmon Resonance Sensors

**Figure 7:**
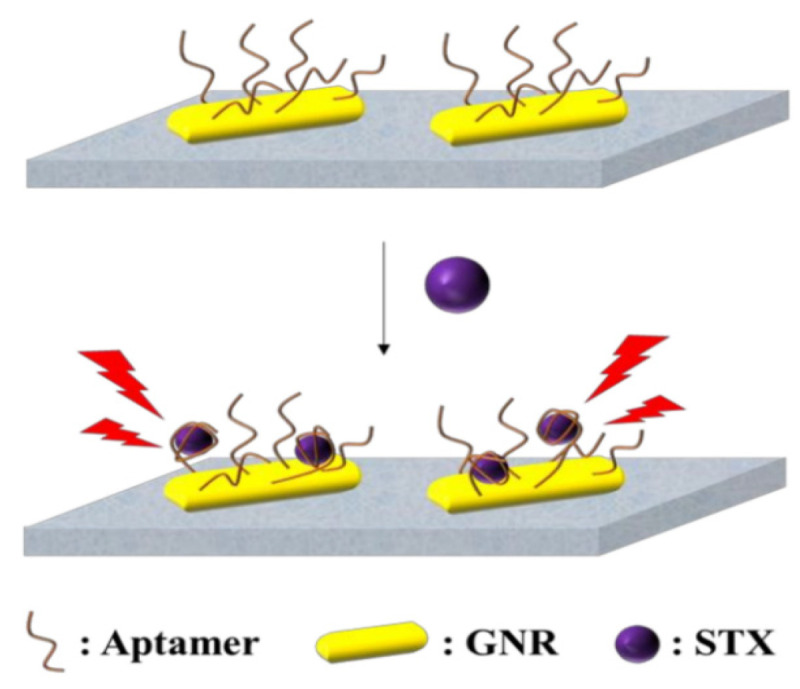
Localized SPR biosensing platform for detection of STX [47].

A surface plasmon resonance (SPR) sensor is based on variations in parameters such as refractive index, resonance angle, mass surface, and so on. Depending on the different mechanisms of action of the combination, different signal displays, and mathematical models of the data, a wide range of analytes, as well as ligand immobilization methods, can be developed for the rapid detection of MBs. SPR sensors have the advantages of high selectivity, high sensitivity, high throughput, real-time monitoring, and low sample consumption [44]. The effective immobilization of marine biotoxin antibodies on the chip directly affects the performance of SPR sensors. The development of a novel STX surface plasmon immunosensor is attributed to optimizing surface treatment on the sensor and improving mixing ratios and times, resulting in increased binding capacity of STX antibodies to the sensor surface [45]. Multichannel and multichannel instrumentation have led to the emergence of more advanced and stable SPR sensors [46]. With the development of marine biotoxin aptamer technology, more and more aptamer biosensors have been reported. For example, Ha et al. developed a localized SPR aptamer sensor, as shown in Figure 7, the biosensor modified gold nanorods via Au-S on a chip, and the STX aptamer was also modified on gold nanorods via Au-S [47]. When STX is present, STX binds to the aptamer, and the localized surface plasmon absorption spectra are shifted to achieve the detection of STX.

These sensors are characterized by their high sensitivity, selectivity, throughput, and ability for real-time analysis with minimal sample use. The effectiveness of SPR sensors heavily relies on the precise immobilization of antibodies, particularly for detecting marine biotoxins like saxitoxin (STX). Recent advancements include the development of multichannel instruments and novel biosensors, such as localized SPR sensors using gold nanorods modified with STX aptamers, allowing for enhanced detection capabilities through shifts in plasmon absorption spectra.

### 4.5. Electrochemical Biosensors

The electrochemical biosensor represents a sophisticated detection technology that utilizes chemical reactions between multiple biorecognition elements to generate electrochemical signals, such as current, potential, and resistance. Renowned for its high sensitivity, rapid response, low cost, and user-friendliness, this sensor can directly analyze the concentration of targeted substances, making it ideal for short-term detection applications. The performance of these biosensors is significantly influenced by the electrode materials used, which facilitate the redox reactions. Nanomaterials have emerged as a focal point of research within this field due to their exceptional electronic properties, catalytic capabilities, extensive specific surface area, and robust biocompatibility, enhancing sensor functionality and accuracy.

For example, Jin et al. investigated a noncompetitive biosensor based on magnetic gold electrode adsorbed palladium-doped graphitic carbon nitride nanosheets catalyzing TMB, which had an LOD of 1.20 pg/mL, fast detection speed, and high sensitivity [48]. Meanwhile, this method uses a gold surface as the immunosensor, which is relatively expensive to produce. In another study, Park et al. used SWV to detect STX in freshwater samples with a linear concentration range of 10 pg mL^−1^ to 1 μg mL^−1^ and an LOD of 4.66 pg mL^−1^ [49]. The sensor was fabricated on a circular microgap electrode, which has the advantage of having a high efficiency (~15 measurements) of the target. 

**Figure 8:**
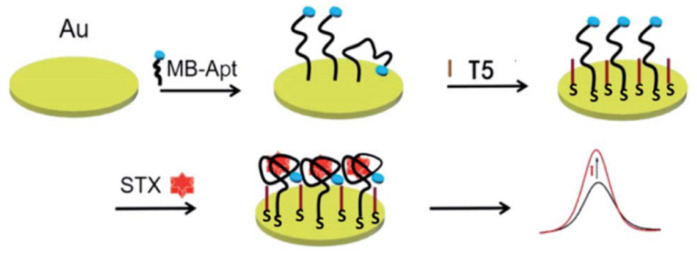
A simple electrochemical aptasensor for detection of STX using MB-Apt for recognition and signal output. Reproduced from [50] with permission from Royal Society of Chemistry.

One of the disadvantages of this sensor is the low feasibility of detecting STX in seawater and seafood samples. In addition, a polyacrylamide hydrochloride-modified aptamer sensor achieved the sensitive detection of STX in the concentration range of 0.50–100.00 nM using amperometric methods [35]. The aptamer and STX are preferably attached near the EIS surface, which accounts for the high sensitivity. The narrow detection range is one of the secondary disadvantages of the system. Furthermore, an amperometric sensor based on the aptamer of STX was developed by Zheng et al., as depicted in Figure 8. In the presence of STX, the methylene blue (MB)-tagged aptamer (MB-Apt) specifically binds to the STX and forms a folded conformation, which brings MB closer to the surface of the electrode and facilitates the transfer of electrons to its surface, which leads to an increase in oxidation current [50]. In electrochemical biosensors, the effective modification of biomaterials directly affects the stability of the sensor, and the study of stable nanomaterials and electrodes is one of the ways to solve these problems.

Electrochemical biosensors use biorecognition elements to produce signals like current or potential, offering high sensitivity and quick response at low costs. They directly analyze target concentrations, primarily in short-term applications. Electrode materials, especially innovative nanomaterials with excellent electronic properties and biocompatibility, are crucial for optimizing reactions. Challenges still remain, such as high production costs for gold-based sensors and limited detection ranges and stability in certain environments.

### 4.6. Piezoelectric Biosensors

**Figure 9:**
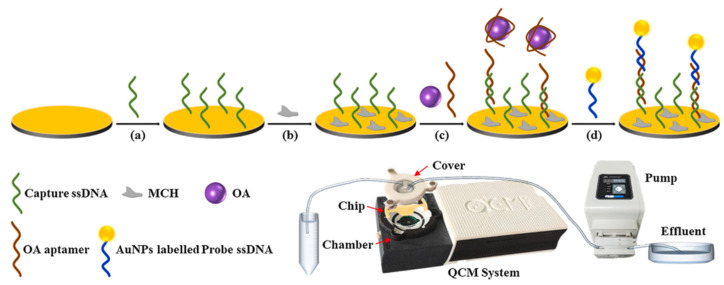
Piezoelectric aptasensor with Au NP amplification for the label-free detection of OA. Reproduced from [51] with permission from Elsevier. (**a**–**d**) represents the order of the procedures.

Piezoelectric biosensors are mainly in the form of a quartz crystal microbalance as a transducer with antibodies, aptamers, specific receptor proteins, etc., bound to the surface of the crystal, which converts the mass change on the surface of the loaded crystal into a resonance frequency change for the detection of MBs. Piezoelectric biosensors are automated, simple, portable, low-cost, and easy to miniaturize, but the long time to establish a baseline, the need for new technical support, and the high price of the instruments have led to fewer applications for marine biotoxin detection. For example, Karaseva et al. immobilized an antibody to OA on the surface of a crystal with an LOD of 1.40 ng/mL [23]. To obtain high sensitivity and introduce signal amplification, Tian et al. designed a Au NP-amplified piezoelectric biosensor as shown in Figure 9, which utilizes OA and Au NPs-DNA to compete for the okadaic acid aptamer on the crystal, and the okadaic acid signal was amplified with an LOD of 0.32 nM [51].

Similar research investigated various molar ratios of OA to bovine serum albumin (BSA) and found that the cross-linked complex exhibited strong adhesion to the gold surface, resulting in an impressive storage lifetime of 38 days. However, the sensor’s detection limit (1.9 μg/mL) and sensitivity were unsatisfactory. By incorporating an antibody-BSA hydrogel, significant improvements in the device’s performance were achieved. Preliminary results indicated a 524-fold increase in the minimum detectable analyte amount and an 80-fold enhancement in sensitivity [52]. In addition, coupled with a Love wave sensor, Zhang et al. developed a novel HepG2 cell-based Love wave biosensor for the sensitive and real-time detection of OA [53]. The results indicated the sensor’s ability to respond to varying cell densities and OA concentrations, with a detection range of 10–100 μg/L. This biosensor, combined with a portable 8-channel instrument, offers a promising solution for convenient and effective OA screening. Another example is an acoustic assay using a 9 MHz AT-cut quartz crystal resonator, modified with a DNA aptamer that changes frequency upon brevetoxin (BTX) binding [54]. The sensor displayed a concentration-dependent frequency shift, with an LOD of 220 nM for BTX, below the maximum residue limit for food. Tested in spiked mussel samples, the sensor showed high specificity without interference from other compounds, indicating its utility for screening mussels for BTX in the food industry.

Despite their advantages of being automated, portable, and low-cost, challenges include a lengthy baseline establishment, reliance on new technical support, and high instrument costs. Innovative approaches like gold nanoparticle amplification have significantly enhanced sensitivity and detection limits. Research has also shown the potential for improved storage stability and sensitivity by modifying detection surfaces and integrating advanced sensor types like Love wave sensors, expanding applications in food safety and environmental monitoring.

## 5. Conclusions

The increasing utilization of marine resources has underscored the pressing issue of marine biotoxin contamination, which has attracted widespread attention in recent years. Safeguarding both human health and marine ecosystems necessitates the vigilant monitoring and detection of marine biotoxins (MBs). Currently, the biosensor detection method stands as a pivotal method for marine biotoxin detection, offering high sensitivity and specificity, thereby serving as an effective tool in safeguarding marine environments and human health. Although significant strides have been made in marine biotoxin detection technology, practical challenges persist, warranting further investigation into the merits and limitations of novel detection technologies. Moreover, exploring the synergistic application of multiple technologies holds promise for enhancing detection efficiency, accuracy, and food safety protection. Below are some notable trends and challenges for biosensors in MB detection.

(1) Miniaturization and portability: There is a growing trend towards the miniaturization and portability of biosensors, enabling the on-site and real-time detection of MBs. This enables the rapid and automated detection of MBs with minimal sample volumes and processing steps, and facilitates field monitoring and early-warning systems for MB outbreaks.
(2) Multiplexed detection: Biosensors capable of multiplexed detection, i.e., detecting multiple toxins simultaneously, are gaining attention. This trend is driven by the need for the comprehensive monitoring of marine environments and reducing analysis time and costs.
(3) Nanotechnology integration: The integration of nanotechnology into biosensor designs allows for enhanced sensitivity and selectivity. Nanomaterials such as nanoparticles and nanocomposites are being explored for improving the performance of biosensors for marine biotoxin detection.
(4) Surface functionalization techniques: Advances in surface functionalization techniques enable the immobilization of biomolecules (e.g., antibodies, aptamers) onto sensor surfaces, enhancing the specificity and stability of biosensors for marine biotoxin detection.
(5) Integration with IoT and data analytics: Biosensors are increasingly being integrated with the Internet of Things (IoT) and data analytics platforms for real-time monitoring and data analysis. This integration enables the continuous surveillance of marine environments and timely responses to toxin events.
(6) Despite these advancements, biosensors for MB detection face challenges such as cross-reactivity, sample matrix interference, standardization and validation issues, as well as concerns regarding field deployment and reliability. Addressing these challenges while capitalizing on emerging trends will drive further advancements in biosensors for the detection of MBs, ultimately contributing to the improved monitoring and management of marine ecosystems and public health.

## Figures and Tables

**Table 1:** Major toxins and sources of MBs.

Classification of Toxins	Major Toxin	Main Sources of Toxins	References
Peptide toxin	Conotoxin, sea anemone peptide toxins, sea serpent toxins	Taro snails, sea anemones, sea snakes	[24,25,26]
Polyether toxin	Ciguatoxin, rock sand anemone toxin, nudibranch toxin	Algae of the genus verbascum gangbytis, short nudibranchs, and sand group anemones	[11,27,28]
Alkaloid toxin	Tetrodotoxin, saxitoxin, genotoxin	Puffer fish, shellfish, gonyaulax	[29,30]
